# Establishment and characterization of a novel childhood acute lymphoblastic leukemia cell line, HXEX-ALL1, with chromosome 9p and 17p deletions

**DOI:** 10.1186/s12935-019-0834-x

**Published:** 2019-04-29

**Authors:** Yiping Zhu, Rong Yang, Ju Gao, Yanle Zhang, Ge Zhang, Ling Gu

**Affiliations:** 1 Laboratory of Hematology/Oncology, Department of Pediatric Hematology/Oncology, Key Laboratory of Birth Defects and Related Diseases of Women and Children (Sichuan University), Ministry of Education, West China Second University Hospital, Sichuan University, Chengdu, 610041 China; 2 Department of Laboratory Medicine, West China Second University Hospital, Sichuan University, Chengdu, China; 3 Joint Laboratory of West China Second University Hospital, Sichuan University and School of Life Science, Fudan University for Pulmonary Development and Disease, West China Second University Hospital, Sichuan University, Chengdu, 610041 China

**Keywords:** Acute lymphoblastic leukemia, Leukemia cell line, Complex karyotype, Chromosome 9p deletion, Chromosome 17p deletion

## Abstract

**Background:**

Although contemporary chemotherapy has improved the cure rate of childhood acute lymphoblastic leukemia (ALL) to nearly 90%, relapsed/refractory ALL is still a leading cause of tumor-related death in children. To clarify the underlying mechanisms of relapsed/refractory childhood ALL, researchers urgently need to establish novel cell models from patients with relapsed ALL after treatment with contemporary chemotherapy.

**Methods:**

Cell culture technique was used to establish the HXEX-ALL1 cell line from primary B cell precursor ALL (BCP-ALL) cells. Molecular and cellular biological techniques including flow cytometry, polymerase chain reaction (PCR), short tandem repeat (STR) analysis, conventional cytogenetics, and chromosomal microarray analysis (CMA) were used to characterize the HXEX-ALL1 cell line. Nude mice were used for xenograft studies.

**Results:**

A stable ALL cell line, HXEX-ALL1, derived from a 6-year-old boy of Han nationality with BCP-ALL at the second relapse, was established and maintained in culture for more than 18 months. The HXEX-ALL1 cell line was authenticated as being derived from primary leukemia cells based on morphologic, immunophenotypic, cytogenetic and STR analyses and demonstrated tumorigenicity in nude mice. WGS data showed that there were 27,006 novel single nucleotide polymorphisms (SNPs) and 193,951 novel insertion/deletions (InDels) in HXEX-ALL1 cells. Compared with the other BCP-ALL cell lines in use, the HXEX-ALL1 cells have a special karyotype represented by trisomy 8 and 9p and 17p deletions with a multidrug resistance phenotype, especially highly resistant to asparaginase.

**Conclusions:**

The HXEX-ALL1 cell line may prove to be a useful model for the study of relapsed/refractory childhood ALL, particularly for the researches on asparaginase resistance.

## Background

Cell culture has been performed for more than a century [1–3]. Cell lines established from tumors are the most commonly used models in tumor research and have enabled a better understanding of the molecular mechanisms of different tumors and provided the means to develop effective treatment strategies [4]. In addition to primary patient materials and animal models, tumor cell lines are one of the three major models for cancer research [4]. The first human cell line was HeLa, established in 1951 by Gey [5]. In 1955, the first human leukemia cell line was derived from a patient with acute myeloid leukemia (AML) by Osgood and Burke [6]. Unfortunately, this cell line soon became contaminated with HeLa cells [7]. Then, the Raji cell line was established as the first leukemia-lymphoma cell line in 1963 by Pulvertaft [8]. Currently, 637 characterized leukemia-lymphoma cell lines are in use [3]. The leading source of leukemia-lymphoma cell lines is Japan (40%), followed by the USA (26%), and only 4 leukemia-lymphoma cell lines have originated in China [3]. Additionally, there are no pediatric leukemia cell lines from China in the ATCC and DSMZ cell banks.

Acute lymphoblastic leukemia (ALL), a malignancy originating from a B or T cell progenitor, is the most common leukemia in children, accounting for approximately 80% of all pediatric leukemia [9]. Fortunately, due to better risk stratification and the advent of multidrug risk-adapted chemotherapy regimens, improved central nervous system prophylaxis and better supportive care, the cure rates are approximately 90% for pediatric patients with ALL [9–14]. For patients enrolled in the CCLG-ALL 2008 protocol in China, the 5-year survival rate was 85.3% [15]. However, 10–15% of patients still relapsed and had a dismal prognosis [14, 16]. Due to the high morbidity of ALL, relapsed/refractory ALL is still the leading cause of tumor-related death in children [14, 16]. Thus, it is very important to clarify the underlying mechanisms of relapsed/refractory ALL. However, to the best of our knowledge, there are no leukemia cell lines derived from pediatric patients relapsed after treatment with contemporary chemotherapy.

In this study, we reported a novel B cell precursor ALL (BCP-ALL) cell line called HXEX-ALL1, which was established from the Bone marrow (BM) of a 6-year boy with BCP-ALL during his second relapse in our hospital. The cell line was authenticated as being derived from primary leukemia cells and displayed tumorigenicity in nude mice. The cell line had a Complex karyotype (CK; with ≥ 3 structural chromosomal abnormalities) with trisomy 8 and 9p and 17p deletions, and displayed a multi-drug resistant phenotype with highly resistant to l-asparaginase (L-Asp). The phenotypic, genetic and functional properties of this cell line were described following the guidelines for the characterization and publication of human malignant hematopoietic cell lines [17].

## Methods

### Case report

The HXEX-ALL1 cell line was derived from a 6-year-old Chinese boy of Han ancestry with BCP-ALL. The patient was admitted to West China Second University Hospital (Chengdu) in 2016 because of podalgia and hemorrhage under the skin. Physical examination upon admission revealed pale lips and enlarged superficial lymph nodes. Complete blood count revealed a white blood cell count of 22.9 × 10^9^/l with 60% blast cells, hemoglobin level of 105 g/l, and platelet count of 52 × 10^9^/l. BM examination revealed hypercellular marrow with 92% blasts that were negative for peroxidase staining. The primary leukemia cells were positive for CD10, CD19, CD22, cCD79 and HLA-DR, partially positive for CD5, and negative for CD20, sIgM, cIgM, CD2, CD3, CD7, cCD3, CD13, CD33, CD117 and CD34 and were thus categorized as the common B subtype according to the EGIL classification [18]. G-banding analysis of the BM revealed the karyotype 47, XY, +8, del(9p22), del(17p12). FISH analysis demonstrated negative expression of *MLL*, *BCR*-*ABL*, *ETV6*-*RUNX1* and *PDGFRB* fusion genes. Multiple real-time polymerase chain reaction (RT-PCR) analyses indicated negativity for the following fusion genes: *MLL*-*AF4*, *MLL*-*AF6*, *MLL*-*AF10*, *TEL*-*AML1*, *MLL*-*ENL*, *BCR*-*ABL P210*, *BCR*-*ABL P190*, *SIL*-*TAL*, *E2A*-*HLF*, *CALM*-*AF10*, *HOX11*, *HOX11L2*, *SET*-*CAN*, *TEL*-*ABL1*, *TLS*-*ERG*, *NPM*-*ALK* and *E2A*-*PBX1*. The patient received chemotherapy according to the Chinese Childhood Cancer Group ALL 2015 (CCCG-ALL-2015) protocol. The regimen included dexamethasone (DEX), pegaspargase (Peg-Asp), vincristine (VCR), daunorubicin (DNR), cyclophosphamide (CTX), cytarabine (Ara-c), mercaptopurine (6-MP), and methotrexate (MTX). After 19 days of chemotherapy, the proportion of blasts in the BM was reduced to 1%, demonstrating complete remission (CR) and negative minimal residual disease (MRD) (< 0.01%). The patient was classified into a low-risk group. However, he experienced BM relapse after 4 months, and re-induction of chemotherapy led to another CR 1 month later. The re-induction chemotherapy regimen included DEX, mitoxantrone (MTZ), vindesine (VDS), Peg-Asp, MTX, etoposide (VP-16), and Ara-c. Unfortunately, the patient experienced a second BM relapse in 3 months, and this time, chemotherapy did not lead to a CR.

### Cell culture

Primary BM cells were obtained from the patient at the second relapse of ALL. The patient provided informed consent. Mononuclear cells were isolated and separated by Ficoll-Hypaque centrifugation and cultured in a 6-well plate (Corning Inc., Corning, NY, USA) at a density of 6 × 10^6^/ml in RPMI-1640 medium (Gibco, Grand Island, NY, USA) supplemented with 20% fetal bovine serum (FBS; Thermo, Grand Island, NY, USA) and 10 ng/ml rhIL-3 at 37 °C with 5% carbon dioxide (CO_2_). The medium was replaced every 3–5 days depending on the cell growth rate to maintain the cells at a density of 1–3×10^6^/ml. The cells were examined daily under an inverted microscope, and the cell number was determined every 3 days with a standard hematocytometer using trypan blue dye exclusion. After 3 weeks of lag phase, the cell number dramatically increased, and the cell density was adjusted to 0.5–2×10^6^/ml. After 5 weeks, rhIL-3 was omitted from the complete medium, and the FBS concentration was reduced to 10% in the complete culture medium after 7 weeks. For subcloning, 1 × 10^5^ cells were seeded in MethoCult GFH4434 (Sigma, St. Louis, MO, USA) medium in six-well culture plates and incubated for 7–10 days. Colonies were extracted and cultured in RPMI-1640 medium. In this study, other leukemia-lymphoma cell lines, such as NALM-6, CCRF-CEM, and Raji, were purchased from Shanghai Institute Cell Resources Bank. All cell lines were maintained in RPMI 1640 supplemented with 10% FBS, at 37 °C in a humidified 5% CO_2_ in-air atmosphere.

### Cell morphologic assay

Morphological characteristics of live cultured cells were observed under an inverted microscope (Olympus, Tokyo, Japan). Smears of BM and HXEX-ALL1 cells were stained with Wright-Giemsa and observed under an optical microscope (Olympus). Cell ultrastructures were observed under a transmission electron microscope (JEOL Ltd., Tokyo, Japan).

### Immunophenotypic analysis

For the detection of the immunophenotype of the patient sample and HXEX-ALL1 cells, we used antibodies against the following targets: CD34, HLA-DR, CD38, CD117, CD56, CD19, CD20, CD79α, cCD79α, CD10, cIgM, sIgM, TdT, CD7, CD3, cCD3, CD5, CD4, CD8, CD2, MPO, CD33, CD13, CD11b, CD64, CD36, CD14, CD15, CD71, CD61, CD41, CD65, and CD45 (Becton–Dickinson Inc., Franklin Lakes, NJ, USA). Positivity for the antigens was determined using a FACSCalibur flow cytometer (Becton–Dickinson Inc.).

### G-banding analysis

Chromosomes were prepared by a standard method and analyzed by the G-banding technique. Karyotype was determined according to the International System for Human Cytogenetic Nomenclature (ISCN, 2013).

### Chromosomal microarray analysis

Genomic DNA was extracted with a Qiagen DNeasy Blood Kit (Qiagen Inc., Valencia, CA, USA) according to the manufacturer’s instructions. Chromosomal microarray analysis (CMA) was performed using Affymetrix CytoScan HD arrays (Affymetrix Inc., Santa Clara, CA, USA). The data were collected and analyzed using the Affymetrix GeneChip Microarray Instrument System (Affymetrix Inc.).

### Western blotting analysis

Western blotting analysis was performed on lysates obtained from HXEX-ALL1, NALM-6, CCRF-CEM, and Raji cells. Proteins were separated by 15% SDS–polyacrylamide gel electrophoresis and transferred onto nitrocellulose membranes (0.22 μm, Millipore, Billerica, MA, USA). Proteins were visualized by incubation with ECL plus reagent (Millipore). All experiments were independently carried out at least 3 times. The level of β-actin protein was used as a control for the amount of protein loaded onto each lane.

### Ig and TCR arrangement analysis

Immunoglobulin (Ig) and T cell receptor (TCR) gene rearrangements analysis were detected by PCR. Genomic DNA was analyzed using multiplex primers designed against *IgVH*-*A (FR1*-*JH)*, *IgVH*-*B (FR2*-*JH)*, *IgVH*-*C (FR3*-*JH)*, *IgDH*-*A (DH1*-*6*-*JH)*, *IgDH*-*B (DH7*-*JH)*, *Igκ (Vκ*-*Jκ)*, *Igλ (Vλ*-*Jλ)*, *TCRB A*, *TCRB B*, *TCRB C*, *TCRG A*, *TCRG B* and *TCRD.* The PCR mixture included the GoTaq Green Master Mix (Promega, Madison, WI, USA), primer mix and genomic DNA. PCR products were visualized using agarose gels stained with SYBR Green I.

### Short tandem repeat analysis

The identity of the HXEX-ALL1 cell line was checked using short tandem repeat (STR) analysis against a BM sample taken from the patient. DNA was prepared from whole BM and HXEX-ALL1 cells using a Qiagen DNeasy Blood Kit (Qiagen) according to the manufacturer’s instructions. The following 22 highly polymorphic STR loci were tested by multiplex PCR: *Amelogenin*, *CSF1PO*, *D13S317*, *D16S539*, *D5S818*, *D7S820*, *TH01*, *TPOX*, *vWA*, *Penta E*, *Penta D*, *D2S441*, *D2S1338*, *D3S1358*, *D6S1043*, *D8S1179*, *D10S1248*, *D12S391*, *D18S51*, *D19S433*, *D21S11* and *FGA*.

### Whole genome sequencing analysis

The whole genome sequencing (WGS) was conducted according to the BGISEQ-500 protocol. Clean reads were aligned to the human reference genome (GRCh37/HG19) using the Burrows-Wheeler Aligner (BWA).

### Cell growth assay

Cells were cultured in a 6-well round-bottom plastic culture plates (Corning Inc., Corning, NY, USA) at 6 × 10^5^/ml in RPMI-1640 medium with 10% FBS and grown for 8 days. Viable cells were counted using trypan blue (Sigma) staining every day. Td was calculated for cells in exponential growth with the following equation: Td (h) = t×lg2/lg(N_t_/N_0_), where t is the time of continuous culture, N_t_ is the final number of cells, and N_0_ is the initial number of cells.

### Cell viability and in vitro drug sensitivity assay

Cell viability was evaluated by the 3-(4,5 dimethylthiazol-2-yl)-2,5-diphenyltetrazolium bromide (MTT) assay. The chemotherapeutic drugs, Dex, L-Asp, VCR, DNR, Ara-C and MTX, were purchased from Sigma. Cells were cultured in the presence of 6 different concentrations of Dex (0.0001–0.1 μM or 0.01–5 μM), L-Asp (0.001–1 U/ml or 0.01–500 U/ml), VCR (0.1–0.6 nM or 0.2–1.2 nM), DNR (1–50 nM), Ara-C (5–100 nM or 50–800 nM) and MTX (2–20 nM or 5–50 nM) for 48 h, respectively, followed by assessment of cell viability by MTT assay. Drug sensitivity was assessed by the IC_50_, drug concentration that inhibits 50% of cell viability. The IC_50_ was calculated by linear interpolation.

### Cell cycle analysis

For each analysis, 10^6^ cells were harvested and fixed overnight in 70% ethanol at 4 °C. The cells were then washed and stained with 5 μg/ml PI in the presence of DNAse-free RNAse (Sigma). After 30 min at room temperature, the cells were analyzed via flow cytometry (Beckman Coulter Inc., Miami, FL, USA) with the acquisition of 30,000 events.

### Animal experiments

Cultured 1 × 10^7^ HXEX-ALL1 cells were subcutaneously injected into the right flanks of 6-week-old female BALB/c (nu/nu) nude mice, with 0.2 ml of PBS injected into the left flanks as the control (*n *= 6). Tumor size was measured by calipers every 2 days. The approximate tumor volume was calculated using the equation V = (length × width × depth)/2. All animal care was in compliance with the guidelines established by the internal Institutional Animal Care and Use Committee and Ethics Committee of Sichuan University. After the mice were euthanized, the tumor mass was excised, fixed in 10% formalin, and routinely processed for paraffin embedding. Five-millimeter-thick sections were obtained and prepared for standard histopathological examination.

### Statistical analysis

All assays were performed in triplicate, and the data were expressed as the mean value ± SD. One-way ANOVA was used to compare two groups. A *P*-value < 0.05 was considered to be significant.

## Results

### Establishment of the HXEX-ALL1 cell line

A stable and prominent cell population was observed following 8 weeks of culture of primary BM mononuclear cells. The cells were maintained in fresh medium (RPMI-1640 medium containing 10% FBS) replaced at 2- to 3-days intervals. The obtained cells were designated HXEX-ALL1 and made available. The HXEX-ALL1 cells had been in culture for 18 months. The Doubling time (Td) was 26–32 h. The cells proliferated consistently and were negative for EBV, HCMV, HBV, HCV, HIV, and mycoplasma infection based on PCR. The cells could be frozen under standard conditions using 60% RPMI-1640 medium, 30% FBS and 10% dimethyl sulfoxide (DMSO) and successfully revived after storage in liquid nitrogen, with more than 80% viability. The cells maintained the same properties after freezing and thawing.

### Morphological characteristics of HXEX-ALL1 cells

**Fig. 1:**
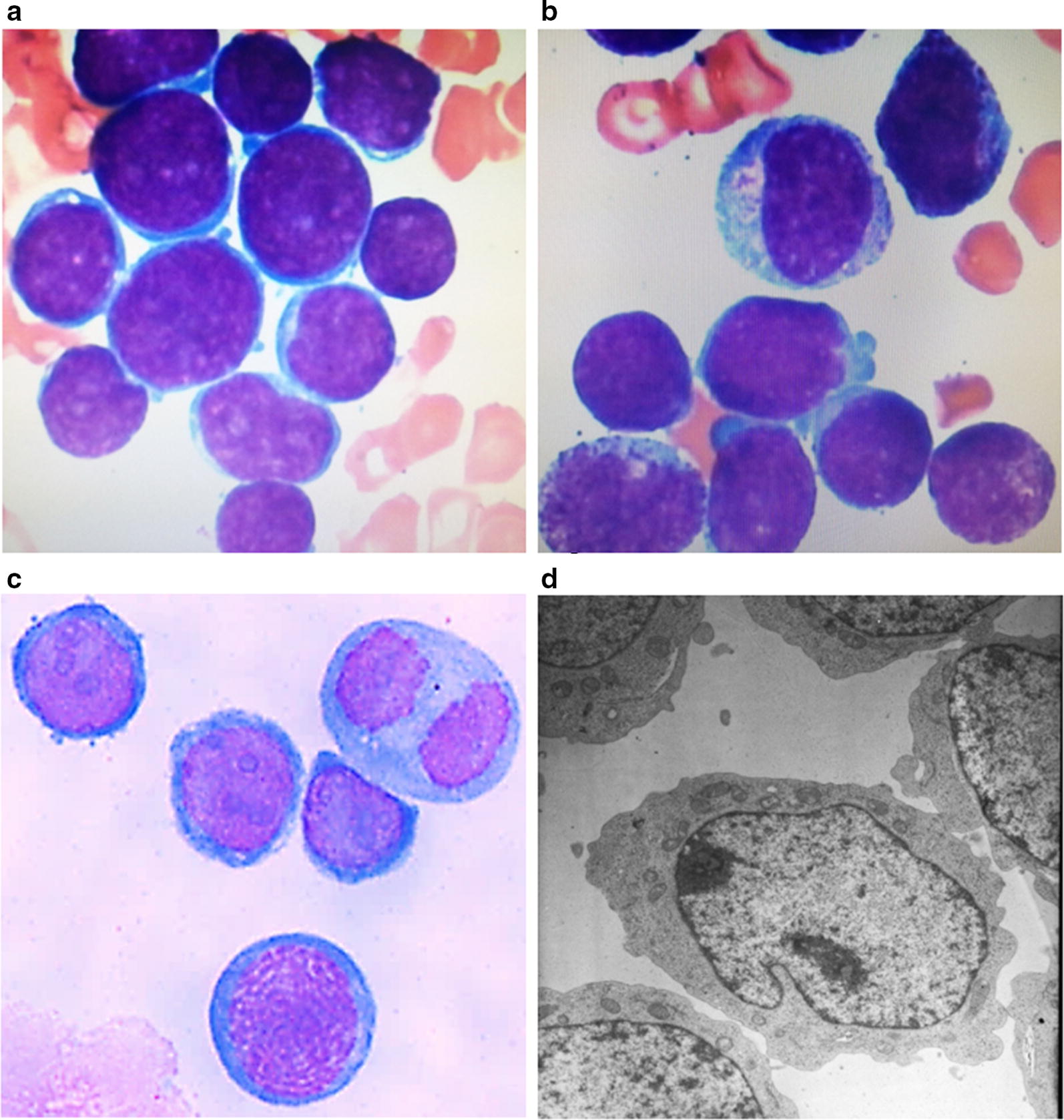
Morphological characteristics of HXEX-ALL1 cells. Wright-Giemsa staining of primary leukemia cells in the BM of the patient. **a** At diagnosis (×1000 magnification) and **b** at second relapse (×1000 magnification). **c** Wright-Giemsa staining of HXEX-ALL1 cells (×1000 magnification). **d** Ultrastructural appearance of HXEX-ALL1 cells (×8000 magnification)

The HXEX-ALL1 cells grew in suspension as single cells or clumps resembled the morphology of lymphoblastic leukemia cells in the patient’s BM smear at diagnosis and second relapse (Fig. 1a, b). The cells were usually round in shape, and the cytoplasm was weakly basophilic with occasional vacuoles; most cells contained only one nucleus that was commonly round; the nucleo-cytoplasmic ratio of these cells was high (Fig. 1c). Mitotic figures were usually observed. Transmission electron microscopy revealed that most cells had a single, large and eccentric nucleus with a fine chromatin network. Few organelles were present in the cytoplasm, except for some mitochondria and ribosomes (Fig. 1d).

### Immunophenotype of HXEX-ALL1 cells

The immunophenotypes of HXEX-ALL1 cells and primary leukemia cells from the patient were virtually identical. The HXEX-ALL1 cells were positive for CD10, CD19, CD22, cCD79α and HLA-DR, partially positive for CD5, and negative for CD20, cIgM, sIgM, CD2, CD3, CD7, cCD3, CD34 and myeloid-, natural killer (NK) cell- and plasmocyte-associated markers. The cells exhibited a common B immunophenotype according to the EGIL classification [18].

### Gross chromosomal alterations in HXEX-ALL1 cells

**Fig. 2:**
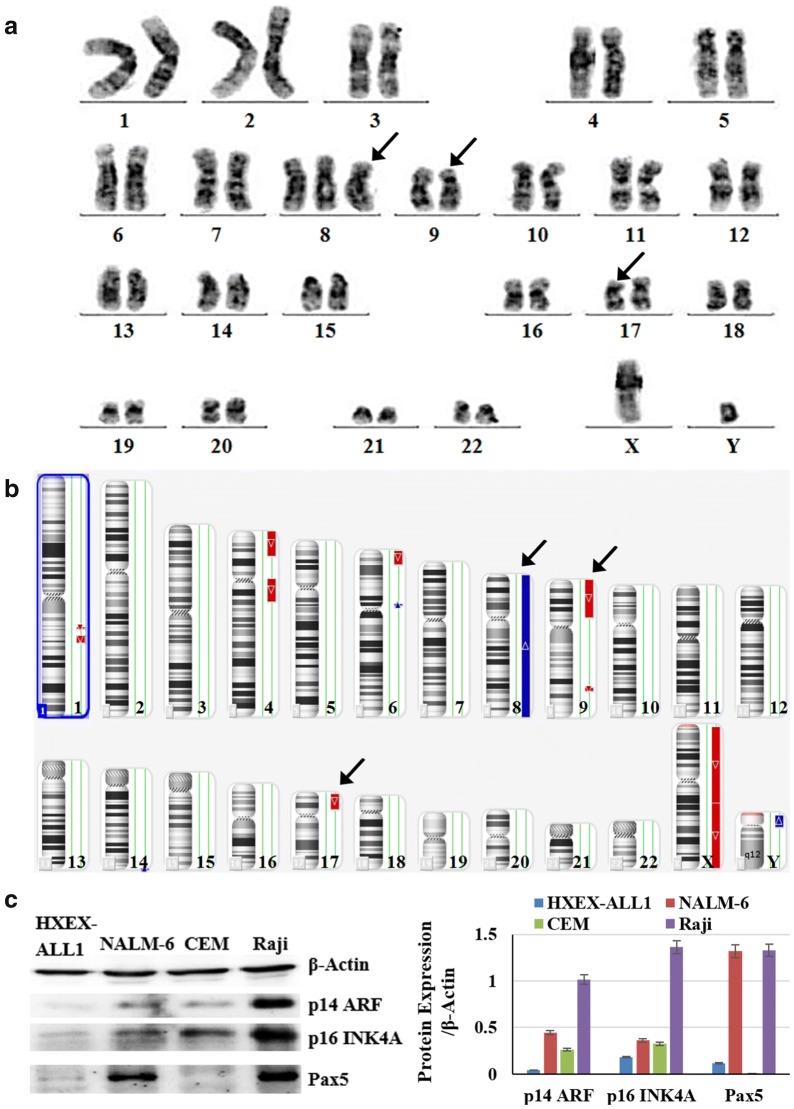
Cytogenetic analysis of primary leukemia cells and HXEX-ALL1 cells. **a** G-banding karyotype of primary leukemia cells. The karyotype according to ISCN (2013) can be described as 47, XY, +8, del(9)(p22), del(17)(p12). Arrows indicate +8, del(9)(p22) and del(17)(p12). **b** CMA of HXEX-ALL1 cells. Arrows indicate the gain of the whole chromosome 8, a 38.56-Mb loss of 9p24.3-p13.1 and a 16-Mb loss of 17p13.3-p11 in all cells. **c** Cells were lysed, and extracts were analyzed by western blotting for p16 ARF, p14 INK4A and Pax5. β-Actin was used as an internal control. Bar graphs show the ratio of protein to β-Actin. Experiments were performed in triplicate

Chromosomal analysis was carried out using the G-banding technique and demonstrated the following karyotype for primary leukemia cells: 47, XY, +8, del(9p22), del(17p12) (Fig. 2a). Because of the failure of G-banding analysis in HXEX-ALL1 cells, CMA was performed after 10 months of culture. The results indicated a gain of chromosome 8, a 38.56-Mb loss of 9p24.3-p13.1 and a 16-Mb loss of 17p13.3-p11 in all HXEX-ALL1 cells (Fig. 2b), consistent with the results of whole genome sequencing. Therefore, the karyotypes of the HXEX-ALL1 cells and primary leukemia cells from the patient were virtually identical. The protein expression levels of p16 ARF, p14 INK4A and Pax5, encoded by the corresponding genes located on chromosome 9p, were low in HXEX-ALL1 cells (Fig. 2c).

### Ig/TCR rearrangements in HXEX-ALL1 cells

Ig/TCR gene rearrangements analysis showed that the HXEX-ALL1 cells harbored the following rearrangements: *IgVH*-*A (FR1*-*JH)*, *IgVH*-*B (FR2*-*JH)*, *IgVH*-*C (FR3*-*JH)*, *IgDH*-*A (DH1*-*6*-*JH)*, *IgDH*-*B (DH7*-*JH)*, *TCRG A* and *TCRD.*

### Identity verification of HXEX-ALL1 cells

Using a multiplex STR system, which enables the detection of unique DNA fingerprints through the genotyping of 22 STR loci, the primary leukemia cells and HXEX-ALL1 cells were found to share 100% identity. These results confirmed that the genetic source of HXEX-ALL1 cells was, in fact, the patient’s leukemia cells (Table 1). The HXEX-ALL1 cell line profile did not match any other cell line profile in the current databases from ATCC, DSMZ or elsewhere.

**Table 1:** STR analysis of HXEX-ALL1 cells, primary leukemia cells and cells from the tumor mass

	HXEX-ALL1 cells	Primary leukemia cells	Tumor cells
Amelogenin	X, Y	X, Y	X, Y
CSF1PO	11, 13	11, 13	11, 13
D13S317	11, 11	11, 11	11, 11
D16S539	10, 11	10, 11	10, 11
D5S818	7, 10	7, 10	7, 10
D7S820	10, 12	10, 12	10, 12
TH01	9, 9	9, 9	9, 9
TPOX	8, 9	8, 9	8, 9
vWA	16, 17	16, 17	16, 17
Penta E	5, 19	5, 19	5, 19
Penta D	9, 11	9, 11	9, 11
D2S441	11, 11.3	11, 11.3	11, 11.3
D2S1338	18, 24	18, 24	18, 24
D3S1358	15, 15	15, 15	15, 15
D6S1043	12, 19	12, 19	12, 19
D8S1179	13, 15	13, 15	13, 15
D10S1248	13, 14	13, 14	13, 14
D12S391	18, 20	18, 20	18, 20
D18S51	13, 23	13, 23	13, 23
D19S433	14, 15	14, 15	14, 15
D21S11	29, 33	29, 33	29, 33
FGA	24, 26	24, 26	24, 26

### Proliferation of HXEX-ALL1 cells

**Fig. 3:**
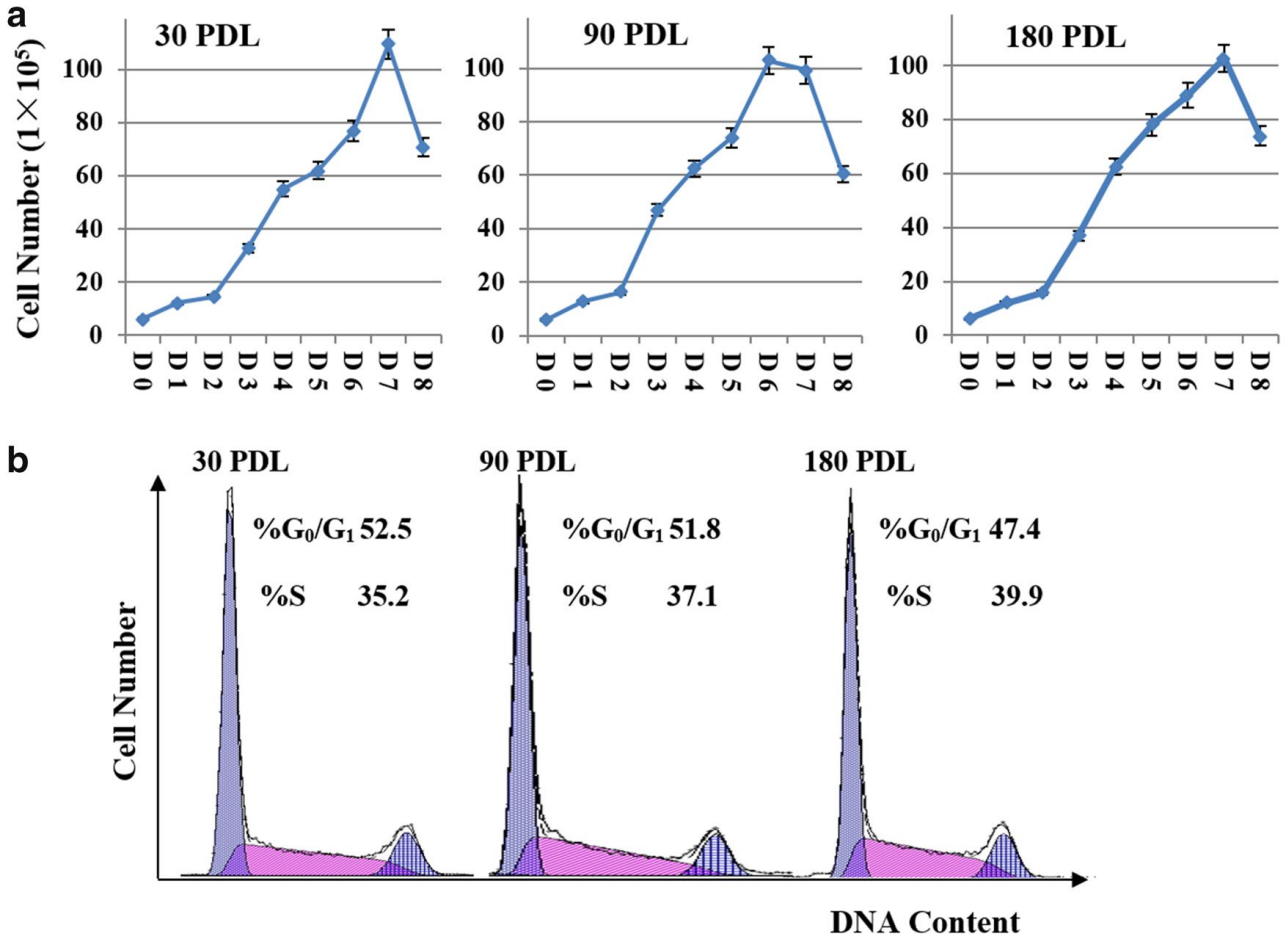
Stable proliferation and division cycle of HXEX-ALL1 cells at different PDLs. **a** Growth curves of HXEX-ALL1 cells at 30, 90 and 180 PDLs. Cells were cultured in a 6-well culture plate at 6 × 10^5^/ml in RPMI-1640 medium with 10% FBS and grown for 8 days. Viable cells were counted using trypan blue staining every day. Cell viability was evaluated by MTT assays. Experiments were performed in triplicate. **b** Cell cycle distribution of HXEX-ALL1 cells at 30, 90 and 180 PDLs. Cells were cultured in a 6-well culture plate at 3 × 10^5^/ml, and cell cycle progression was analyzed by PI staining after 48 h. Experiments were performed in triplicate. The data was analyzed with the MultiCycle Software for Windows (Phoenix Flow Systems, San Diego, CA). The percentage of cells in G_0_/G_1_, G_2_/M and S phase was not significantly different (*P *> 0.05) among different PDLs

We determined the proliferation for 8 days at different cell Population doubling levels (PDLs) of HXEX-ALL1 cells (Fig. 3a). The results revealed that HXEX-ALL1 cells stably proliferated in RPMI-1640 medium containing 10% FBS with a population Td of 26–32 h. The percentage of cells in G_0_/G_1_, G_2_/M and S phase were similar without significant differences (*P *> 0.05) at different PDLs (Fig. 3b), suggesting that the HXEX-ALL1 cells could stably maintain the cell division cycle during continuous culture in vitro.

### In vitro drug sensitivity of HXEX-ALL1 cells

**Fig. 4:**
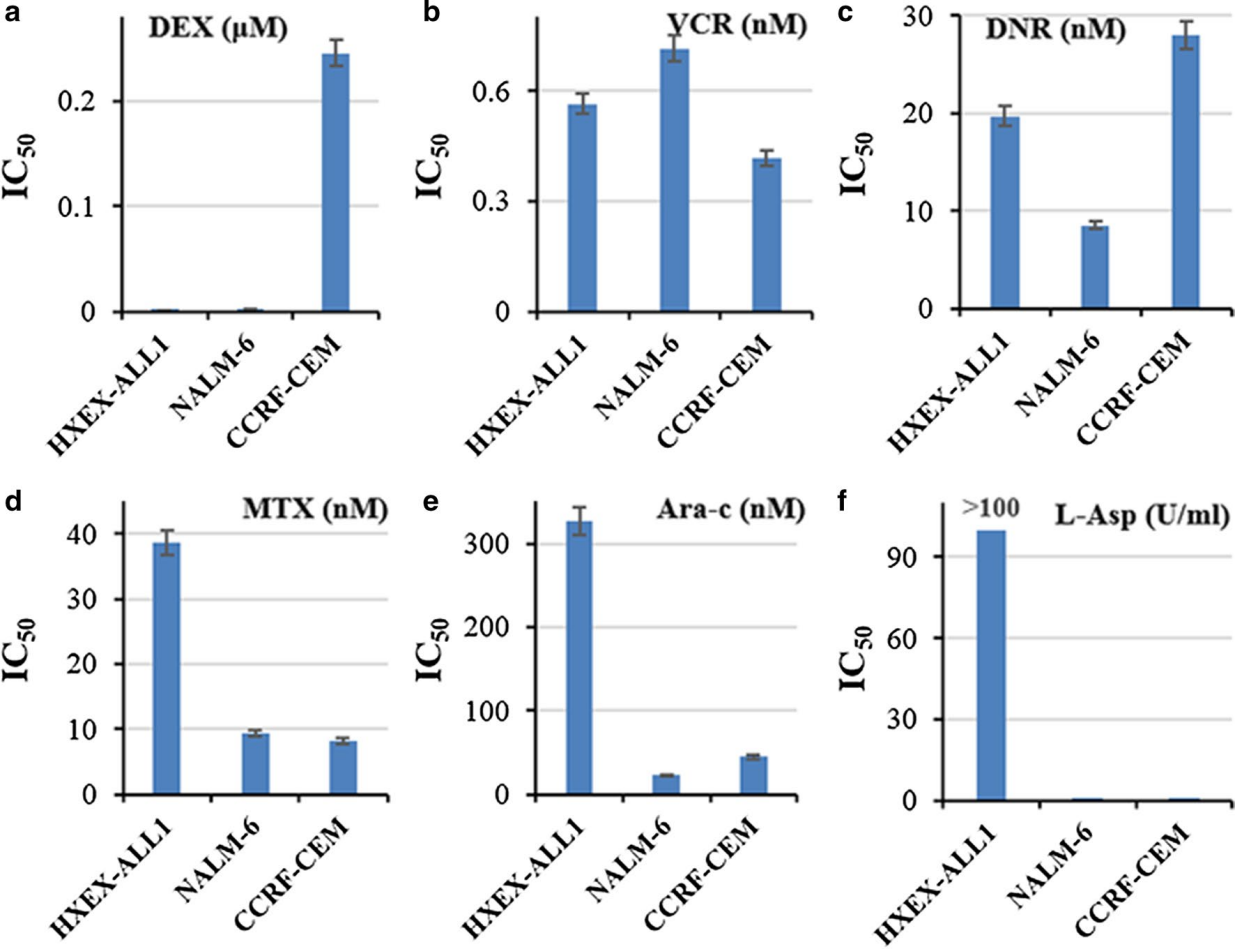
Drug sensitivity of HXEX-ALL1 cells. **a**–**f** IC_50_ of HXEX-ALL1, NALM-7 and CCRF-CEM cells to Dex, VCR, DNR, MTX, Ara-c, and L-Asp. Cells were cultured with increasing concentrations of different drugs for 48 h. Cell viability was evaluated by MTT assays. The IC_50_ values were calculated by linear interpolation. Experiments were performed in triplicate

HXEX-ALL1 cell line was constructed from a relapsed BCP-ALL patient, and might be resistant to chemotherapeutic drugs. The cell lines, HXEX-ALL1, NALM-6 (BCP-ALL) and CCRF-CEM (TCP-ALL), were tested for their sensitivity to the 6 drugs most commonly used in the treatment of pediatric ALL. The results showed that HXEX-ALL1 cells were sensitive to Dex and VCR (Fig. 4a, b). Compared to NALM-6, HXEX-ALL1 were resistant to DNR (2.3 fold) (Fig. 4c), MTX (4.1 fold) (Fig. 4d), Ara-c (14.2 fold) (Fig. 4e), and highly resistant to L-Asp (> 409.8 fold) (Fig. 4f).

### WGS data of HXEX-ALL1 cells

The WGS analysis resulted in a mean of 2,359,620,422 clean reads. There were 3,357,771 single nucleotide polymorphisms (SNPs), 847,741 insertion/deletions (InDels), 3093 copy number variations (CNVs) and 6896 structural variations (SVs). Among them, 27,006 SNPs and 193,951 InDels were novel when compared with dbSNP and the 1000 Genomes Project databases.

### The tumorigenicity of HXEX-ALL1 cells

**Fig. 5:**
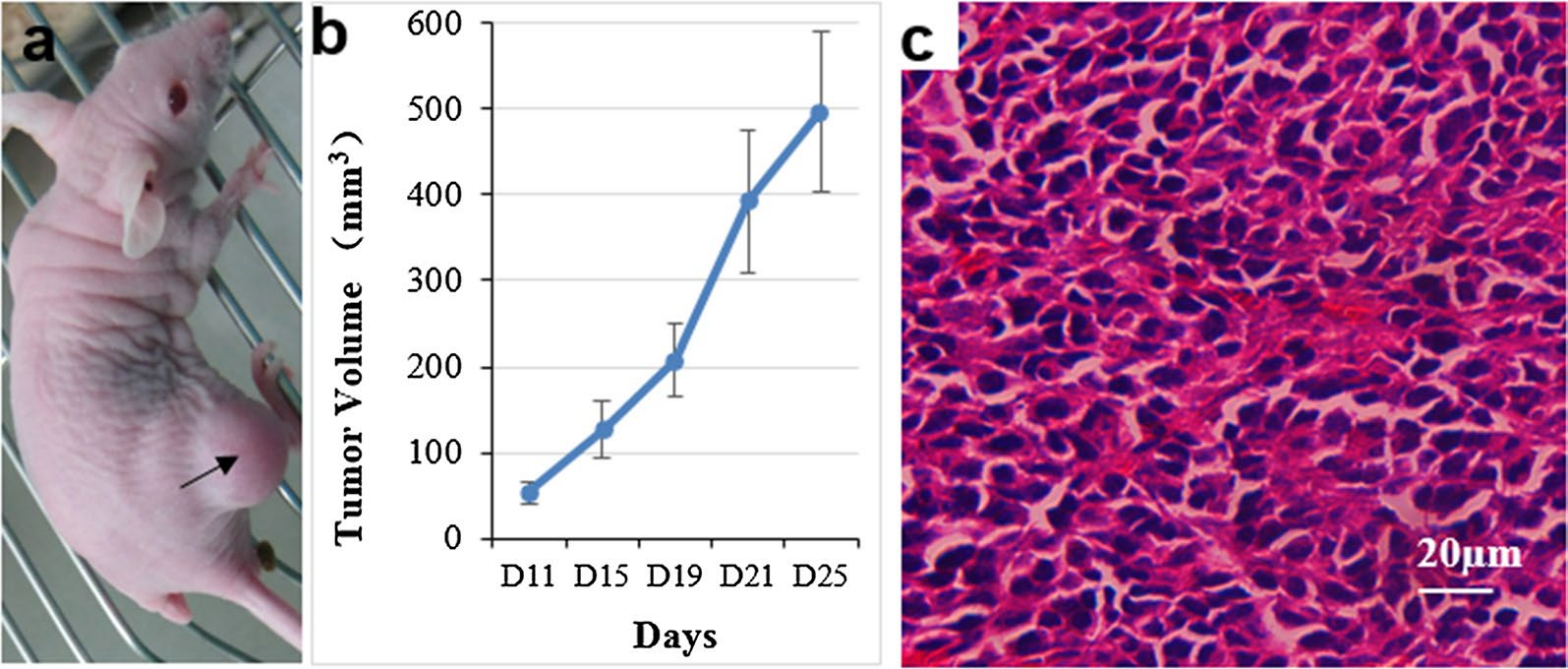
Tumorigenicity of HXEX-ALL1 cells. **a** Subcutaneous tumor mass in nude mice (arrow indicated). **b** The growth curve of subcutaneous tumors in nude mice (n = 3, age 5–6 weeks). All animal procedures were carried out in accordance with the guidelines established by the internal Institutional Animal Care and Use Committee and Ethics Committee guidelines of Sichuan University. **c** HE staining of the tumor showed that the tumor mass was composed of leukemia cells and blood vessels. Original magnification: ×200 magnification

To determine the tumorigenicity of HXEX-ALL1 cells, we subcutaneously injected 1 × 10^7^ cells into female BALB/c (nu/nu) nude mice (n = 6). After 25 days, subcutaneous tumors were observed in 3 mice, with a mean volume of 497.1 ± 111.5 mm^3^ (n = 3) (Fig. 5a, b). Hematoxylin and eosin (HE) staining indicated that the tumor masses were composed of leukemia cells (Fig. 5c). The results of STR analysis indicated that subcutaneous tumors were derived from HXEX-ALL1 cells (Table 1).

## Discussion

Improvements in the prognosis of childhood ALL represent one of the most successful stories in modern medicine. Moreover, this improvement has taken place not only in developed countries but also in developing countries because conventional chemotherapy is inexpensive and readily available worldwide [12–14]. The major reason for the improvement in prognosis is the reduction in the risk of relapse as a result of risk-directed chemotherapy [12–14]. However, the management of relapsed and refractory ALL is still difficult due to chemoresistance. Despite efforts to intensify re-induction strategies, including allogenic hematopoietic stem cell transplantation, the Overall survival (OS) rates for patients with relapsed ALL remain between 25 and 40%, highlighting the need for researches on new promising drugs, such as immunotherapeutic agents [19]. However, the mechanisms underlying ALL progression, relapse and resistance remain elusive. Thus, the establishment of new cell lines derived from patients who are refractory to contemporary chemotherapy would facilitate the elucidation of the biological properties of relapsed and refractory ALL and development of novel therapeutic strategies. However, the establishment of a hematopoietic malignancy cell line from the BM is still challenging.

Currently, there are 637 characterized leukemia-lymphoma cell lines, and among them only 4 are from China. The majority of these cell lines were established from 1977 to 1997 [3]. Contemporary chemotherapeutic protocols have improved the cure rate of pediatric ALL to nearly 90% in the last 2 decades [9–14]. Nevertheless, to the best of our knowledge, there are no cell lines derived from patients who are resistant to contemporary chemotherapeutic protocols, highlighting the need for novel cell lines derived from relapsed and refractory patients. Fortunately, we established a novel cell line, HXEX-ALL1, from a Chinese boy of Han ancestry with a second relapse of BCP-ALL. All major features of the HXEX-ALL1 cell line are summarized in Table 2. As authenticated by the morphologic, immunologic, cytogenetic and STR analyses, the HXEX-ALL1 cells are derived from the patient’s primary leukemia cells. Thus far, the HXEX-ALL1 cell line has been continuously grown in culture for over 18 months (over 400 PDL) with a Td of 26–32 h and has maintained stable proliferation and cytogenetic features. Therefore, the HXEX-ALL1 cells are representative of patient leukemia cells. More importantly, compared with the other BCP-ALL cell lines in use [20, 21], the HXEX-ALL1 cells have a special karyotype represented by trisomy 8 and 9p and 17p deletions and display a multidrug resistant phenotype with highly resistant to L-Asp. Moreover, WGS data showed that there were 27,006 novel SNPs and 193,951 novel InDels in HXEX-ALL1 cells.

**Table 2:** Summary of HXEX-ALL1 cell features

Feature	HXEX-ALL1 cells
Clinical characteristics	
Patient	6-year-old male
Diagnosis	BCP-ALL
Treatment status	At second relapse
Specimen site	Bone marrow
Year of establishment	2017
Cell culture characteristics	
Culture medium	90% RPMI-1640 + 10% FBS
Growth pattern	Suspension as single cells or clumps
Doubling time	26–32 h
Subculture	1:4
Maximum cell density	10 × 10^6^ cells/ml
Optimal cell density	1 ~ 3×10^6^ cells/ml
Cryoperservation	60% RPMI-1640 + 30% FBS + 10% DMSO
Morphology	Medium-sized spheroid morphology
Viral status	Negative for EBV, HCMV, HBV, and HIV
Contamination	Negative for mycoplasma
Authentication	Yes (by STR analysis, cytogenetic analysis, and immunoprofiling)
Immunophenotypic characteristics	
B cell	CD10+, CD19+, CD22+, cCD79α+, CD20−, cIgM−, sIgM−
T cell	CD2−, CD3−, CD5+, CD7−, cCD3−
Myelocytic	CD13−, CD33−, CD117−
Progenitor	CD34−, HLA-DR+
Ig/TCR rearrangements	*IgVH*-*A, IgVH*-*B*, *IgVH*-*C*, *IgDH*-*A*, *IgDH*-*B*, *TCRG A* and *TCRD* rearrangements
Gross chromosomal alterations	
CMA	47, XY, +8, del(9p24.3-p13.1), del(17p13.3-p11)
Drug sensitivity	Sensitive to Dex and VCR
	Resistant to DNR, MTX, Ara-c
	Highly resistant to L-Asp
Functional characteristics	
Clonality	Yes
Tumorigenicity in nude mice	Tumor masses in 3/6 nude mice

Gross chromosomal alterations are a hallmark of ALL [22]. Cytogenetic risk is used to classify patients into low-, intermediate- or high-risk groups. As defined by the suggestion of the Subspecialty Group of Pediatric Hematology, of Chinese Medical Association (2014), patients with a Philadelphia (Ph) chromosome (encoding the *BCR*-*ABL1* fusion gene) and MLL rearrangements are classified into the high-risk group, patients with *E2A*-*PBX1* rearrangements in the intermediate-risk group, and other patients into the low-risk group [23]. The list of genetic alterations in childhood ALL that are associated with the risk of relapse continues to grow. Ph chromosome, *BCR*-*ABL1* like with *IZKF1* and *JAK2* alterations, MLL rearrangements, hypodiploidy (< 44 chromosomes or DNA index < 0.81), and iAMP are assigned as unfavorable characteristics [24]. In contrast, hyperdiploidy with trisomy 4 and 10 and *ETV6*-*RUNX1* fusion are designated favorable genetic factors [24]. The results of G-banding analysis in the patient showed that the primary leukemia cells had 3 gross structural chromosomal abnormalities, namely, trisomy 8, and 9p and 17p deletions, but no unfavorable characteristics at diagnosis. The patient was classified into the low-risk group at initial CR. Unfortunately, the patient experienced relapse 4 months after the CR and achieved a second CR after re-induction chemotherapy but then relapsed again after 3 months.

A CK with ≥ 3 structural chromosomal abnormalities is not listed among the criteria of risk classification for childhood ALL. However, a CK with ≥ 4 structural chromosomal abnormalities is predictive of poor outcomes in adult AML patients without favorable or adverse aberrations [25]. A study of 428 adult patients with Ph-negative ALL demonstrated that a CK is an adverse prognostic factor independent of the MRD status, but the definition of CK should include ≥ 5 chromosomal abnormalities [26]. The MRC UKALL XII/ECOG E2993 study enrolled 1522 adult patients with ALL and demonstrated that patients with a CK comprising ≥ 5 chromosomal abnormalities had an inferior prognosis [27]. A study of 79 pediatric patients with ALL indicated that a CK with ≥ 3 chromosomal abnormalities should be considered a poor prognostic factor in childhood ALL [28]. Nevertheless, the poor prognosis associated with CK remains controversial; some other studies have indicated that a CK does not have a significant impact on either OS or event-free survival in ALL [29–31].

Additionally, deletion of 17p, a segment containing the *TP53* gene, is an independent risk factor in adult patients with AML and chronic lymphocytic leukemia (CLL) [25, 32, 33]. In adult ALL, abnormal 17 alterations including 17p deletions may be a risk factor in T cell precursor ALL (TCP-ALL) [34, 35] but not in BCP-ALL [27]. In childhood BCP-ALL, 17p deletions predict a poor prognosis and a higher rate of relapse in patients without abnormalities in *ETV6*–*RUNX1* or hyperdiploidy [36].

Deletion of 9p is a common chromosomal alteration detected since 1983 [37]. Chromosome 9p contains numerous tumor-associated genes, such as *PAX5* at 9p13.2, *CDKN2A*, *CDKN2B*, *MTAP, IFN*, *MLLT3*, *PTPLAD2* at 9p21.3, and *JAK2* at 9p24.1 [38, 39]. However, the impact of 9p deletions on ALL outcomes is contradictory. Deletion of 9p is associated with favorable prognosis in adult patients with Ph-negative ALL [27] and unfavorable prognosis in patients with Ph-positive ALL [40]. In childhood ALL, some studies have shown that 9p deletions have no association with outcomes [36, 41, 42]. In contrast, other studies have indicated that 9p deletions predict poor prognoses and risk of relapse in both BCP- and TCP-ALL [43–46]. A recent study on relapsed childhood ALL indicated that 9p deletions are associated with relapse [47]. In the refined risk classification of childhood ALL, favorable genetic features include *ETV6*-*RUNX1*, hyperdiploidy, lack of deletion of *IKZF1*, *CNKN2A/B*, *PAR1*, *BTG1*, *EBF1*, *PAX5*, *ETV6* or *RB1*, isolated deletions affecting *ETV6/PAX5/BTG1*, and deletions of *ETV6* with an additional deletion of *BTG1/PAX5/CDKN2A/B*; other genetic features are classified as risk factors [48]. *PAX5* and *CDKN2A/B* genes are located on chromosome 9p. The protein expression levels of Pax5, p16 ARF and p14 INK4A, encoded by *PAX5* and *CDKN2A/B* genes, were low in HXEX-ALL1 cells compared with the NALM-6 and Raji cells. Most recently, novel subtypes of ALL based on diverse *PAX5* alternation, including PAX5alt, PAX5 p.Pro80Arg, or PAX5-plus, were reported [49–51]. PAX5-plus patients, with *PAX5* p.Pro80Arg as hotspot and biallelic genomic alterations, had a favorable treatment outcome in trials of population-based German study cohorts [50]. In contrast, the outcome for PAX5 p.Pro80Arg in children treated on St. Jude Total Therapy protocols was unfavorable [49]. PAX5alt patients, with homozygous or heterozygous *PAX5* alterations, including rearrangements, sequence mutations and focal intragenic amplifications, had an intermediate outcome in the Children’s Oncology Group AALL0232 study [49]. In this study, PAX5 p.Pro80Arg patients had an intermediate outcome also [49]. Therefore, 9p deletion might play a complex and paradoxical role in relapsed ALL patients.

Trisomy 8 is reported in 10–15% of adult AML patients, 9–12% of adult ALL, and 2–10% of childhood ALL, mainly as a part of hyperdiploid karyotypes or CKs with structural abnormalities [25, 26, 52, 53]. The prognosis of AML patients with trisomy 8 depends on whether it occurs as an isolated abnormality or is accompanied by other aberrations [54]. Trisomy 8 alone had no significant impact on the prognosis of adult ALL [27]. However, the relationship between trisomy 8 and prognosis in childhood ALL is still unknown [54]. Increased attention should thus be paid to the impact of CK, trisomy 8, 9p and 17p deletions on the prognosis of ALL.

Furthermore, accompanying with the special karyotype, HXEX-ALL1 cell line shows a multidrug resistant phenotype with resistant to DNR, MTX, Ara-c, and L-Asp. The outstanding characteristic of HXEX-ALL1 is that the IC_50_ to L-Asp was higher than 100 nM after 48 h treatment, and were 409.8- fold more resistant than NALM-6 cells. Over the past 50 years, Asp has become a key chemotherapeutic agent for ALL and has led to a substantial improvement in cure rates, especially in children [55, 56]. Asp resistance plays a main role in the treatment failure of ALL [56]. The underlying mechanism of ALL cells resistant to Asp remains unclear. However, the researches on Asp resistance are far fewer than the researches on glucocorticoid resistance. One important reason is that there are some glucocorticoid resistant ALL cell model, but few Asp resistant model.

## Conclusions

To the best of our knowledge, HXEX-ALL1 is the first BCP-ALL cell line derived from a pediatric patient who relapsed after receiving appropriate contemporary chemotherapy. The novel BCP-ALL cell line resembles the primary leukemia cells at diagnosis. Moreover, the novel cell line has 27,006 novel SNPs and 193,951 novel InDels. Compared with the other BCP-ALL cell lines in use, the HXEX-ALL1 cells have a special karyotype represented by trisomy 8 and 9p and 17p deletions with multi-drug resistant phenotype, especially highly resistant to L-Asp. Thus, HXEX-ALL1 may be a valuable cell model for further investigation on the mechanisms and potential targeted therapy of relapsed/refractory ALL, particularly for those developed a dominantly resistant to Asp. Meanwhile, the HXEX-ALL1 cell line represents a useful model to explore the impact of 9p and 17p deletions on the pathogenesis and prognosis of ALL and to develop new target drugs.

## Abbreviations

ALL: acute lymphoblastic leukemia
AML: acute myeloid leukemia
BCP-ALL: B cell precursor ALL
BM: bone marrow
CK: complex karyotype
CMA: chromosomal microarray analysis
Ig: immunoglobulin
PDL: population doubling level
TCR: T cell receptor
Td: doubling time
